# Mediterranean fruit fly population phenological patterns are strongly affected by elevation and host presence

**DOI:** 10.1038/s41598-024-56714-x

**Published:** 2024-03-12

**Authors:** Vasilis G. Rodovitis, Eleni Verykouki, Kostas D. Zarpas, Stella A. Papanastasiou, Cleopatra A. Moraiti, Nikos Patronis, Nikos T. Papadopoulos

**Affiliations:** Laboratory of Entomology and Agricultural Zoology, Department of Agriculture, Crop Production and Rural Environment, University of Thessaly, Volos, Greece

**Keywords:** *Ceratitis capitate*, Tephritidae, Population monitoring, Population dynamics, Elevation, Host availability, Capture probability, Ecology, Agroecology, Climate-change ecology, Population dynamics

## Abstract

The Mediterranean fruit fly (medfly) (*Ceratitis capitata*, Diptera: Tephritidae), is an extremely polyphagous pest that threatens the fruit production and trading industry worldwide. Monitoring *C. capitata* populations and analysing its dynamics and phenology is considered of outmost importance for designing and implementing sound management approaches. The aim of this study was to investigate the factors regulating the population dynamics of the *C. capitata* in a coastal and semi-mountainous area. We focused on effects of topography (e.g. elevation), host presence and seasonal patterns of ripening on the phenological patterns considering data collected in 2008. The experimental area is characterized by mixed fruit orchards, and Mediterranean climate with mild winters. Two trap types were used for population monitoring. The female targeted McPhail type and the male targeted Jackson type. Traps were placed in farms located at different elevations and landscape morphology (coastal and semi-mountainous areas). The main crops included citrus, apples, peaches, plums, pears, figs, quinces and apricots. Adult captures were first recorded in May, peaked in mid-summer and mid-autumn and almost ceased at the end of the season (January 2008). Captures in the coastal areas preceded that of highlands by 15 days. Most of the adults detected during the fruit ripening of late stone fruit cultivars (first peak) and citrus (second peak). The probability of capturing the first adults preceded almost three weeks the peak of adult captures either considering the elevation or host focus analyses. The results provide valuable information on the seasonal population trend of *C. capitata* in mixed fruit Mediterranean orchards and can support the set-up of IPM systems in areas with various landscapes and different hosts throughout the fruit growing season.

## Introduction

Fruit flies (Diptera: Tephritidae) are considered among the most destructive fruit pests worldwide. The population dynamics of tephritids and phenology patterns are related to several biotic and abiotic factors^1–4^. Temperature, humidity and host availability are strongly related with altitude and particularities of landscape in each area generating temporal and spatial gradients that regulate population densities and dispersion of fruit flies^5–7^ and that of other insects^8^. Despite the wealth of information on seasonal patterns of fruit fly population, the effects of host trees, ripening seasons and elevation have not been elucidated in detail^4,9–16^.

The Mediterranean fruit fly (medfly), *Ceratitis capitata* (Wiedemann) (Diptera: Tephritidae), is a tropical multivoltine and extremely polyphagous frugivorous pest that can infest fruits of more than 300 different plant species^17,18^. Originated in eastern parts of the sub- Saharan Africa^19^, mostly throughout the intensive international trade of various host fruits and the intense human mobility^20^ and its plastic and adaptive biological properties^21^, *C. capitata* managed to successfully disperse to almost all continents (Africa, Europe, Middle and Near East, several islands of the Indian Ocean, South and Central America, western Australia and Pacific islands) ^22,23^. In Europe, it has been present throughout the Mediterranean countries for the past couple of centuries. Detections of *C. capitata* populations have been recently reported in northern parts of the Mediterranean Sea and Central Europe (e.g. Austria, Germany, Switzerland), in black sea (e.g. Romania and Ukraine) and continental Balkan countries such as Romania and Serbia^24–26^. Population modelling studies based on climate change scenarios and ever-increased knowledge on thermal biology limits and response to challenging environments predict a further northward expansion of the geographic range of this species in the near future^27–30^.

Insect richness, evenness and population abundance decline with elevation increase due to impoverishment of habitat suitability^8,31^. Elevation influences temperature, humidity and host occurrence in each area, factors that are strongly correlated with the phenology of insects in general^31^ and tephritids in particular^10,14,15,32–34^. The adaptation of the insects to higher elevations usually entails reduced number of generations, extending time intervals between the compliance of lifecycles or diapause events and changes on the thermal tolerance, longevity and sexual maturity^8,31,35,36^. Studies revealed that *C. capitata* can be found at elevations up to 2000 m from sea level^9,10,16,32,35,37^. It seems that survival, persistence and thriving of *C. capitata* populations at such high elevations is accomplished through longer life cycles (extended adult longevity, slower reproduction rates), patchy seasonal occurrence and survival in favorable microclimate habitats^11,34,35^. The altitudinal effect on *C. capitata* ecology and behavior may provide valuable information regarding the response of the pest population to climate change and plastic adaptation to challenging environments^31^.

The small and often mixed fruit orchards that prevail in several European countries constitute favorable environments for the development and persistence of *C. capitata* populations. The structure of the landscape in such an environment including ripening sequence, dispersion and abundance of key hosts, cultivation practices and slopes may determine epidemic configuration of populations and infestation rates of commercial crops^38^. Because of its long lifespan and reproduction in the wild^39,40^ a single *C. capitata* female can potentially infest both early and late ripening fruit species and/or cultivars, such as apricots or nectarines and apples or citrus, respectively^4,7,41^. On the other hand, succeeding generations can breed in sequentially ripening hosts. Early ripening hosts are important for the foundation of the first summer generation and late ones may serve as overwintering sources and refugia for females and larvae respectively. Abundance and dispersion of key hosts determine population growth in an area. Infestation rates as well as adult population densities in key hosts provide essential elements to construct predictive models and design sound management interventions. Although plenty of trapping systems used in the past for *C. capitata* monitoring, the prediction of first adult occurrence during the fruiting season, which can guide farmers’ management strategy, still needs further examination. Likewise, the effect of host dispersion, abundance and ripening season on the population dynamics of *C. capitata* has received rather limited attention^38,42–44^.

The aim of the current paper was to analyze the population dynamics of the *C. capitata* in a mixed fruit area that includes pome, prune and citrus fruit. We focused our analysis on effects of key hosts, temperature and the elevation as a major landscape element. Hence, the scope of this study was (a) to appraise the phenology and seasonal biology of *C. capitata* in mixed fruit orchards, (b) to depict the effect of elevation and host on phenology and (c) to utilize new analytical tools to thoroughly understand population dynamics of *C. capitata* and present seasonal population trends.

## Results

### Effect of elevation on the phenological patterns (Study 1)

**Figure 1:**
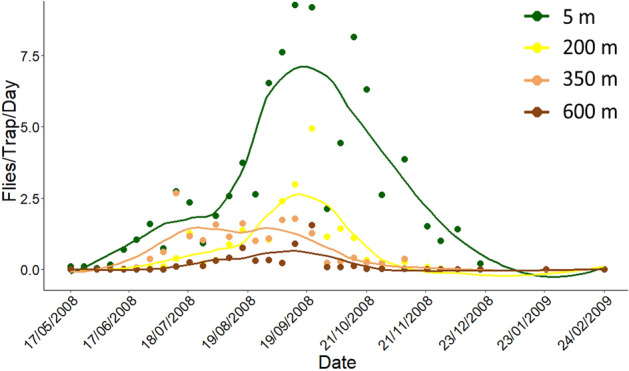
Seasonal patterns of adult *C. capitata* captures at different elevations in the slopes of mount Pelion during 2008. LOESS curves (Locally Estimated Scatterplot Smoothing curves) were fitted capture data at 5, 200, 350 and 600 m elevations.

Seasonal patterns of *C. capitata* captures at four different elevations are given in Fig. 1. The first captures were recorded during May. Captures peaked in September and October and ceased in early January. The total number of captured adults in all plots and traps was 8,194. The highest captures per trap per day were reported at the lowest elevation (Tables S1 and S2). Female captures exceeded those of males in all elevations (Wald *x*^2^ = 547.33, df = 1, *p* < 0.001) (Table S2). Both the active period of trapping as well as the number of captured adults followed an altitudinal gradient. The first captures were reported in the traps deployed at 5 m elevation at the beginning of May while that at 600 m in the middle of July (Fig. 1). A GEE negative binomial model revealed a decrease by 4% on total captures, male and female captures (*p* < 0.001, *p* < 0.001 and *p* < 0.001 for total, male and female captures, respectively) for every 10 m increase in elevation (Table S1).

**Figure 2:**
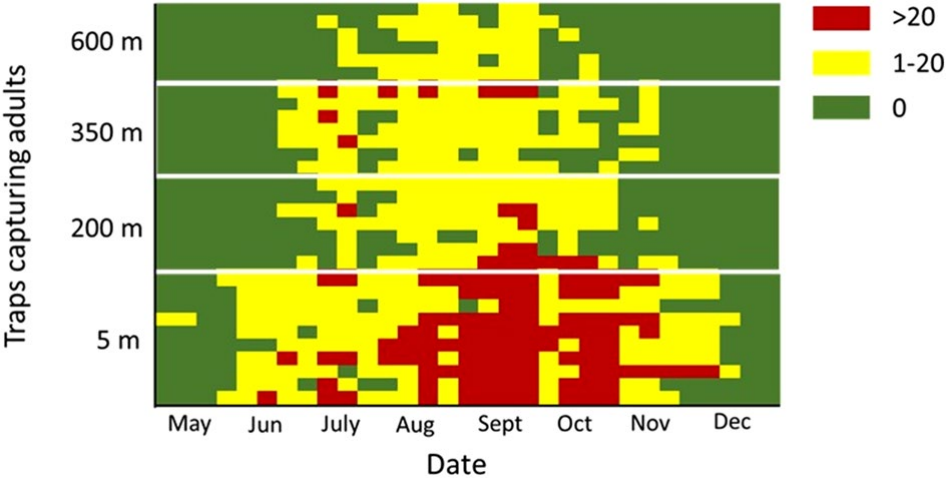
Event history diagram depicting captures per trap placed at different elevations of the mount Pelion during 2008. Each horizontal line represents a single trap and colors stand for adult captures (Red: > 20 flies/week, yellow: 1–20 flies/week, green: 0 flies/week).

To depict the seasonal pattern of *C. capitata* captures in the four different elevations, an “event history” diagram was constructed (Fig. 2). On the lowest elevation (5 m), the first captures were recorded in May, continued throughout the summer, peaked (> 5 captures per trap per day) in both September and October and declined in December, reaching a whole activity period of eight consecutive months. At 200 and 350 m elevation levels, the period of adult captures was shorter (five months), started in June and ended in November, while reached the highest rates in September. The situation was quite different at the highest elevation of 600 m where population activity was reported from the beginning of July until the end of October and reached its peak in September, completing a short flight period of four months.

**Figure 3:**
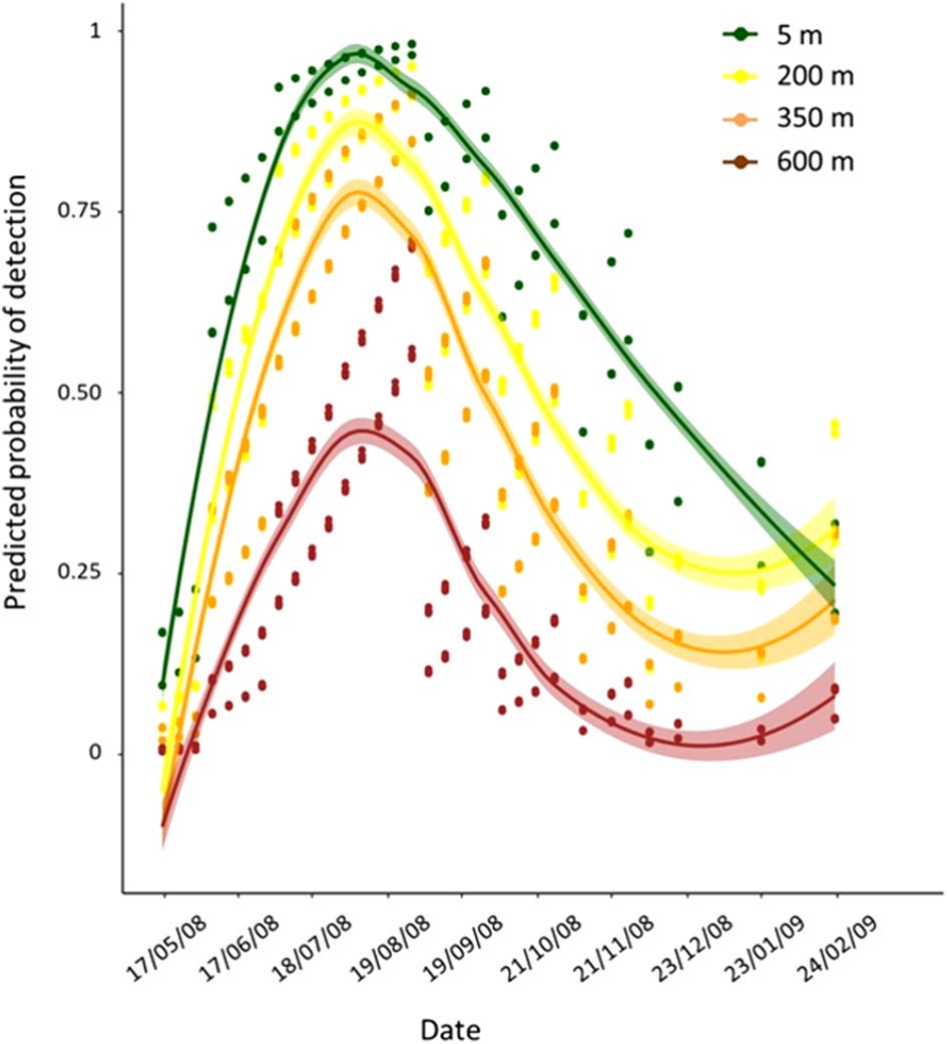
Seasonal patterns of predicted probability of detecting an adult for the different elevations. LOESS curves (Locally Estimated Scatterplot Smoothing curves) were used to smooth patterns for each elevation (5, 200, 350 and 600 m) during the capturing period during 2008.

The effect of elevation on the probability of detecting an adult using the given trapping strategy during the monitoring period is shown in Fig. 3. Both elevation and date were significant predictors of adults’ detection (Wald *x*^2^_elevation_ = 23.20, df = 1, *p* < 0.05) (Wald *x*^2^_date_ = 17.44, df = 1, *p* < 0.05). The detection probability was higher from early July until the end of September in all elevations and increased as elevation decreased. While the predicted probability of detection was more than 0.75 for a period of 5 months (from June until October) at the lowest elevation (5 m), it was lower than 0.4 throughout the season at the higher elevation (600 m) (Fig. 3).

### Effect of tree host and temperature on adult captures (Study 2)

**Figure 4:**
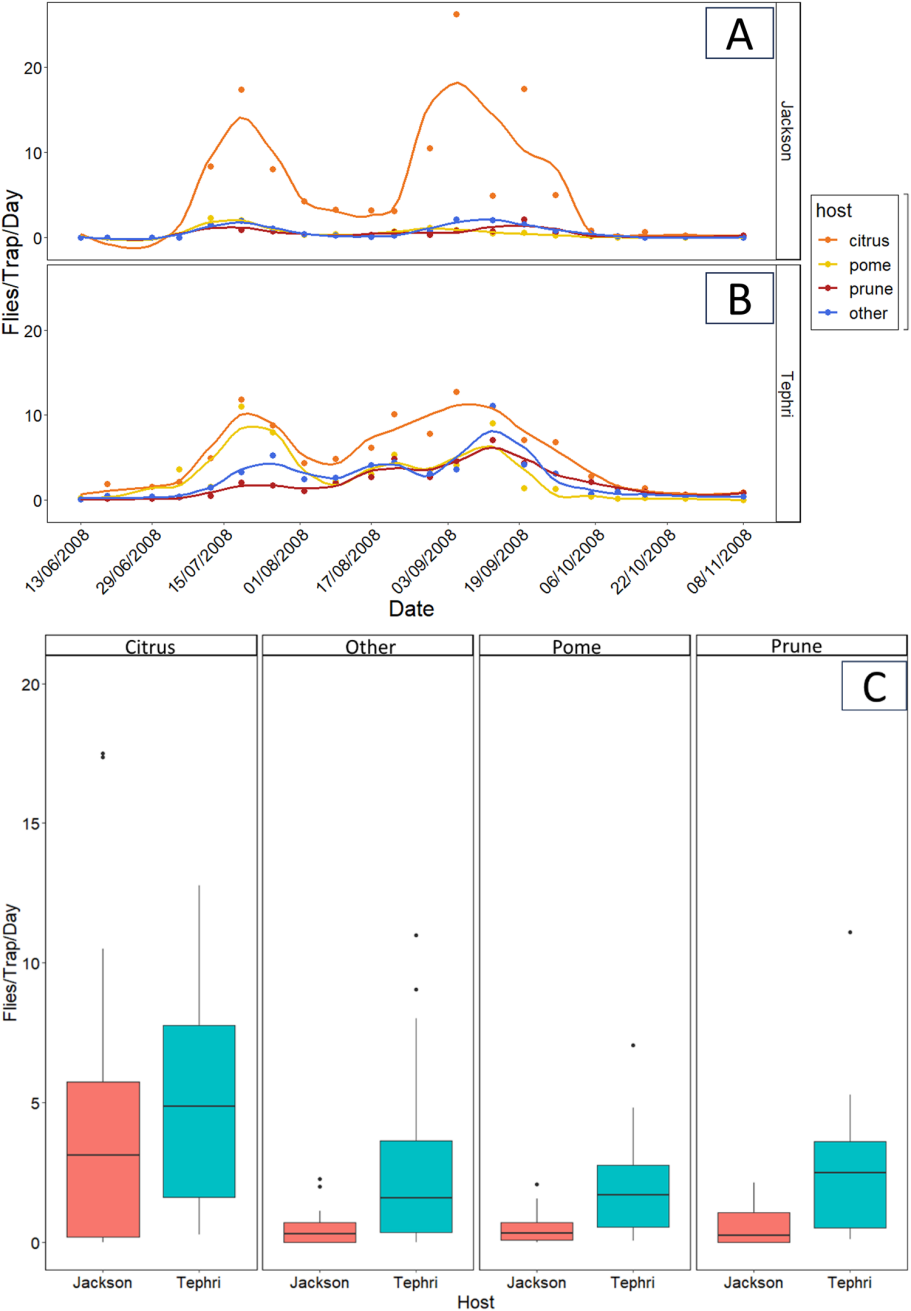
Adult captures per trap per day with LOESS curves (Locally Estimated Scatterplot Smoothing curves) for both Jackson (**A**) and Tephri (**B**) traps, and box plots (**C**) of captured adults per trap per day for both Tephri and Jakson traps for citrus, other, pome and prune during 2008.

Seasonal pattern of *C. capitata* captures in different hosts are given in Fig. 4. In all hosts the first adults were captured in June. Two picks of captures were recorded, the first in July and the second in September (Fig. 4A, B). The total number of adults captured was 29,564 considering all traps and hosts. We used a separate model for each trap type. Both host (Wald *x*^2^ = 91.15, 53.20, df = 3, *p* < 0.001 for Jackson and Tephri respectively) and temperature (Wald *x*^2^ = 80.36, 25.10, df = 1, *p* < 0.001 for Jackson and Tephri respectively) affected adult captures throughout the season. Captures differed among hosts as the temperature increased (Wald *x*^2^ = 79.57, 39.34, df = 3, *p* < 0.001 for Jackson and Tephri respectively). The highest captures reported in citrus in both Jackson (*p* < 0.05) and Tephri (*p* < 0.05) traps (Tables S5 and S6) (Fig. 4A, B). The captures in Jackson traps started in July, peaked in September and declined in November (Fig. 4A). Captures in Tephri traps followed a similar pattern, the only difference being that the capturing period started earlier in June and declined with higher captures in November (Fig. 4B). The highest captures were reported in citrus, while no differences were reported among prunes, pomes and other hosts (Table S5 and S6) (Fig. 4C). Most of the captures reported in citrus, while the captures in prunes, pomes and other hosts were relatively low in the Jackson traps (Fig. 4C) (Table S6).

**Figure 5:**
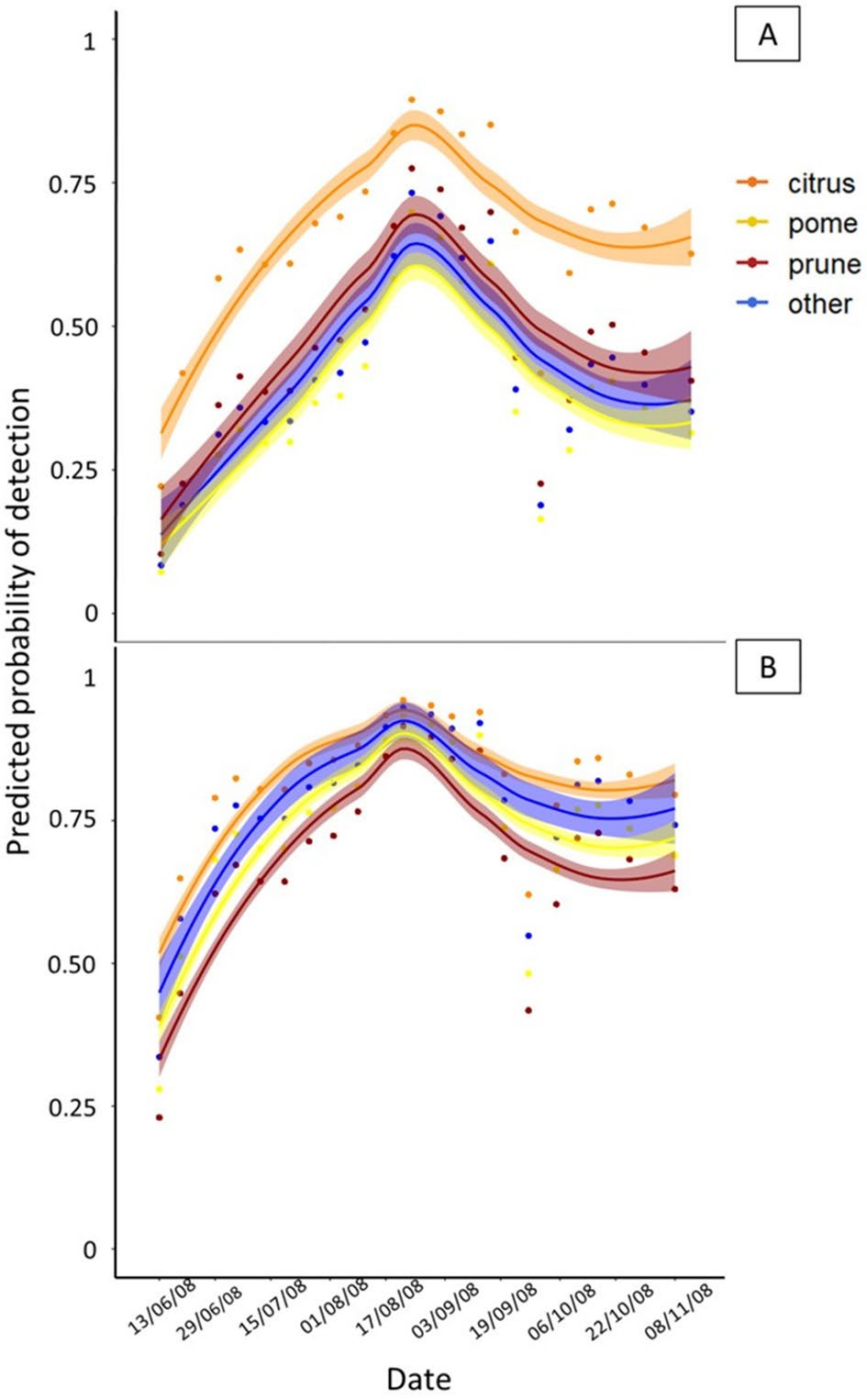
Predicted probability of detecting an adult for the different hosts (citrus, pome, prune, other) for both Jackson (**A**) and Tephri (**B**) traps, as well as LOESS curves (Locally Estimated Scatterplot Smoothing curves) during the capturing period from 2008.

The effect of host on the probability of detecting an adult using the given trapping system during the monitoring period is shown in Fig. 5. Host, date and temperature were significant factors on adults’ detection in Jackson traps (Wald *x*^2^_host_ = 19.37, df = 3, *p* < 0.001) (Wald *x*^2^_date_ = 63.74, df = 1, *p* < 0.001) (Wald *x*^2^_temperature_ = 38.17, df = 1, *p* < 0.001) (Fig. 5A). Although date and temperature significantly affected the adult detection in Teprhi traps as well, (Wald *x*^2^_date_ = 54.30, df = 1, *p* < 0.05) (Wald *x*^2^_temperature_ = 88.04, df = 1, *p* < 0.05) host was not a significant predictor (Wald *x*^2^_host_ = 4.16, df = 3, *p* = 0.25). Indeed, captures occurred at approximately the same period in all hosts (Fig. 5B). The interaction between the date and the host was not significant as well. The predicted probability of detection was higher from the beginning of August until the mid of September in all host types and traps. In citrus the probability of detecting adults remained higher than 0.5 from early July until the end of November (almost 5 months in a row) in the Jackson traps, while in all the other hosts the same period did not exceed 3 months in a row (Fig. 5A) (Table S5). In the beginning of the capturing period, the probability of detecting an adult using the Tephri trap was higher (> 0.25) than that of Jackson (< 0.25), which was higher than 0.25 only in citrus hosts (Fig. 5).

## Discussion

Our results show the activity of adult *C. capitata* flies, revealed by trap captures, lasted in the area of Lechonia, Volos Greece from the end of May until the end of December. Regardless of elevation and host type following the first detection in May, two peaks in adult captures occurred: the first in the middle of July and the second in September. Both host phenology and elevation regulate population patterns of *C. capitata* in the area. The probability of detecting adults decreased with an increase in elevation and followed similar seasonal patterns. However, population dynamics followed different patterns in the different elevations. It seems that different factors determine detection efficacy and population density levels. The probability of capturing adults was higher at lower elevations throughout the season. Interestingly, it remained at high levels even at the higher elevation of 600 m from mid-July until mid-September, while in 5 m it exceeded 0.5 from June until November (approximately 5 months). As expected, the hosts considered in the current study (citrus, pome, prunes and other marginal hosts) exerted strong effect on adult captures. Although citrus was reported as the major host for the pest monitoring in the given area, the other two host types, prunes and pomes should also be considered especially at higher elevations where citrus are scarce or absent. The Tephri trap was more efficient early in the season regardless of the host type. However, Jackson traps capture more adults when deployed on citrus trees. Considering the demographic approaches followed to analyze trapping data (e.g. event history diagrams) we gained a better insight on the seasonal and host-related patterns of capture probability, which may be important for managing fruit flies in an area.

The seasonal population fluctuation of *C. capitata* in the area of Pelion is similar with other areas of southern Europe and western Asia, especially locations with Mediterranean climatic profile. Two populations peaks, in mid-summer and in mid-autumn periods, were reported in Croatia^45^, Italy^15,46^ Montenegro^47^, Spain^48^, Turkey^49^, Israel^50^, and other parts of Greece, particularly in Thessaloniki, northern Greece^44^, Chios island^51^ and Crete^52^. Usually, the peaks of *C. capitata* population depend on several different factors, such as host ripening period, host abundance and dispersion, temperature, elevation and landscape^1–6,53^.

Across the Mediterranean region, stone fruits and especially apricots and peaches are commonly harvested from early June until the end of August (period of the first peak), followed by the plethora of citrus species that are present from early September until the end of November reaching ripening or harvesting stage (period of the second peak) in the same coastal areas. Pome fruits succeed and overlap in ripening with prunes at the end of summer and citrus at the beginning of autumn. Both peaches and citrus are listed within the most favorable hosts for *C. capitata* contributing to population increase and are correlated with population peaks^15,38,44,45,51,54–56^.

Evaluating the efficacy of two traps, one targeting males and one capturing most females^38,57–59^ in relation to host, date of capture and temperature, we found out that Tephri performed better than Jackson capturing more flies in all hosts and detecting earlier the first captures regardless of the host. The Jackson performed better in citrus species compared to the other hosts. Our results are in line with those of Papadopoulos et al. (2003) demonstrating that McPhail type traps (e.g. Tephri) baited with food attractants are more suitable for early detection of *C. capitata*. Tephri trap captures were not affected by the host in our study during the season, in contrast to Papadopoulos and colleagues above, in which the host maturation stage was found to affect detection efficacy especially early in the season. In our case we reported large number of captures and absence of variation between the hosts throughout the season on both traps except of these deployed on citrus that were not present in the area where Papadopoulos and colleagues conducted the above study^38,43^. Citrus species considered as one of the key hosts for *C. capitata* population growth and their presence in the orchard may justify high captures in both trap types and increase the probability of detecting the adults^51,60^. Citrus hosts, especially mandarins, sweet oranges and bitter oranges, are important resources for *C. capitata* along the Mediterranean region. The fruits remaining on the tree during the winter served as overwintering refugia and also as the first fruit available for infestations at the beginning of the season^51^. Additionally, the maturation of citrus fruits early in the autumn and the presence of mature fruits until the end of January explains the uninterrupted trapping period of adult medflies. Although Jackson traps baited with trimedlure are by far the most preferred for population monitoring of *C. capitata*^43,44^ they capture only males and hence provide no information for detection and population dynamics of females. The inclusion of the labor demanding McPhail type traps increases the detection efficacy and accuracy in depiction of population trends in an area since both sexes are sampled^38,58,59^. Our results support the use of the two trap types fix to accuracy of population monitoring of *C. capitata*^38,43,58,60^.

*Ceratitis capitata* population abundance and trapping probability are both reduced with an increase in elevation and reduction of host occurrence and dispersion. Elevation has been described as an essential factor affecting the population growth and abundance of fruit flies in general^6,16,61^ and *C. capitata* specifically^4,5,12,13,34,49^. Elevation is strongly connected with host availability and temperature, environmental factors which are contributing to establishing favorable microclimates for *C. capitata* growth and dispersion^5^. Increase in elevation usually drives a decrease in temperature which differentiates host availability and ripening period^4,5,38^. *Ceratitis capitata* survival and abundance in higher elevations are dramatically affected by the continuous, low temperatures in the beginning of the fruiting season that spans the end of spring ^44,48,62^. In addition, it is well known that tropical and subtropical fruits including citrus, that are among the most favorable hosts of *C. capitata,* are not cultivated in higher elevations^16,63^; hence, *C. capitata* survival depends mainly on other hosts, such as pome (apples and pears) or stone (apricots and peaches) fruits^44,64^. Increased winter survival of *C. capitata* has been reported in coastal Mediterranean areas^65,66^ where a plethora of favorable hosts such as citrus are cultivated. However, in higher elevations the lack of citrus and the presence of other late ripening hosts, such as pome fruits^38,42^, in combination with low temperatures during spring delay the population growth and lead to reduced adult captures.

Our data clearly demonstrate that peaks of capture probability precede that of population density by approximately one month adjusting for elevation and host. Trapping probabilities determine the number of traps capturing at least one adult during the observation period. Several studies focus on early detection of first adults since the correlation between population peaks and higher infestation levels seems to be doubtful^42,49,51^. Early detection is quite relevant in low prevalence or pest free areas and may be related with major management decisions that affect not only control but fruit trading, extirpation, containment and eradication activities^21^. Bringing new tools to analyze capture data, such as the event history approach followed in the current study, may shed important new light on the detection of invasive fruit flies and the population monitoring under low population densities. In addition, predicting the exponential population growth before reaching the peak^67^ is extremely important to prevent the destructive infestations. Connecting the dots between trapping probabilities and population densities revealed by flies per trap per day may contribute to develop more accurate population modeling for specific areas and even farms. Apparently, additional factors regulating *C. capitata* population growth and dispersion such as temperature, humidity and landscape structure and fragmentation should be considered as well^21,68^.

## Conclusion

Our study highlights the effects of elevation and host presence on the population dynamics and dispersion patterns of *C. capitata* in central Greece. Both elevation and host availability significantly influence the phenology of the fly. Through comprehensive analyses of *C. capitata* captures at different elevations and in various hosts in mixed European orchards, we lay the groundwork for developing and implementing more effective management strategies to protect fruits. The differences in seasonal shifts of population occurrence at different elevations alter the optimal timing for pest management applications targeting *C. capitata* in each elevation. The differentiation in population dynamics among hosts refines the seasonal focus on each host, optimizing pest management applications to enhance fruit protection. The results of both studies could be used on modeling approaches focused on predicting population occurrence and dynamics in pest-free areas where similar hosts and topography are present. Similar modeling approaches could utilize these results to evaluate simulations of population dynamics in such landscapes on farm or wider level. The new data analysis tools employed in our study to predict population occurrence before peak events may be adapted for early detection, prevention, and successful pest management applications for *C. capitata* and other pests.

## Materials and methods

### Field sites

**Figure 6:**
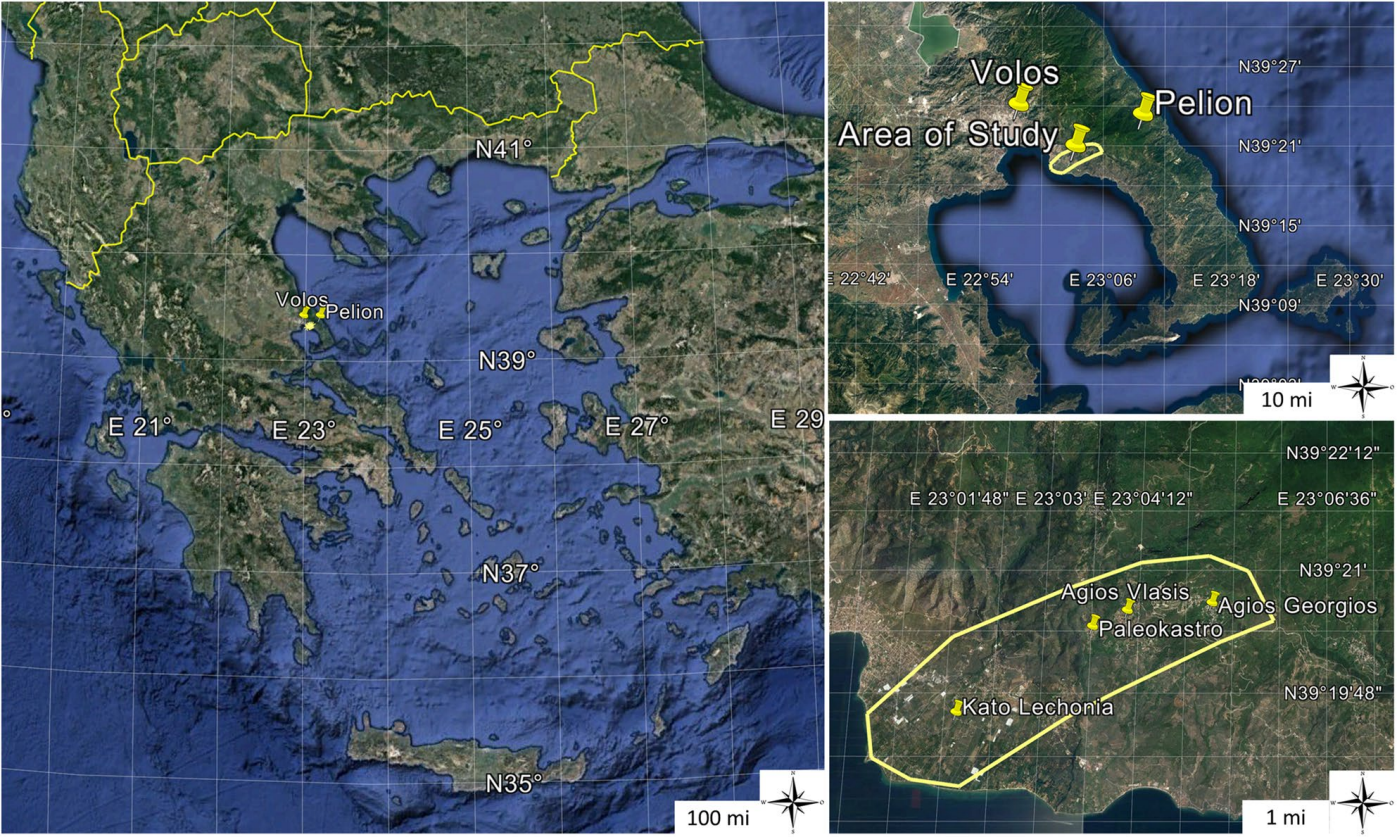
Geographic location of mount Pelion (left) and the four locations (right) where adult traps for monitoring *C. capitata* population were placed.

Two studies were conducted in 2008, in one coastal and three semi-mountainous areas located in mount Pelion, Magnesia, Greece where *C. capitata* population was monitored. For the first study, four areas located at different elevations were selected (Fig. 6): (a) Kato Lechonia (3–5 m), (b) Paleokastro (198–217 m), (c) Agios Vlasis (331–345 m), (d) Agios Georgios (612–630 m). In the second study, population monitoring, focused on different hosts, was conducted in the coastal plain of Kato Lechonia.

### General description of study area

Magnesia county has a rather diverse geography with Pelion Mountain meeting the Aegean Sea to the east and the coastal fertile plain of Kato Lechonia to the west. A total of approximately 320 ha in Pelion are cultivated with stone—pome fruits and citrus^69^. For the needs of the two studies, an area of approximately 1500 ha in total was selected, stretching from the coast to the slopes of the mount (Fig. 6). Several important *C. capitata* hosts are cultivated in the area including citrus fruits (bitter oranges, mandarins, sweet oranges), pome fruits (apples, pears, quinces), stone fruits (apricots, peaches, plums) and others (figs and pomegranates). The fruiting period begins in May with apricots, cherries, followed by peaches, pears figs in summer, apples and quinces early in autumn and mandarin, sweet and bitter oranges later in autumn and winter. Conventional, biological and abandoned farms are present in the area as well as scattered summer houses and small towns with numerous backyards. Climatic data were obtained from the Greek national database “Meteo” (http://meteosearch.meteo.gr/) referring to the city of Volos. Detailed description referring to the climatic profile of the area and the climatic data used in this study (Figs. S1 and S2) are provided in the Supplementary materials file (Section S1).

### Mediterranean fruit fly monitoring

McPhail type traps (International Pheromone McPhail trap (IPMT) or Tephri McPhail trap) that capture both males and females and Jackson traps capture almost exclusively males, were used to monitor the population dynamics of *C. capitata*. In the McPhail type traps, the insects were retained in 200 ml of water containing 0.1% of propylenglycol. In the Jackson traps insects were captured on the sticky surface of the trap. Traps were inspected every week, and all captured insects were counted and removed, starting from early May until the end of February 2008. Trimedlure and Biolure dispensers (placed in Jackson and McPhail type traps respectively), were replaced every 3 and 6 weeks respectively. Traps were placed 1.5–1.8 m above the ground in the shaded part of the canopy^44,48^.

#### Effect of elevation on adult captures (study 1)

To monitor the adult population at different elevations in the first study, a total of 30 McPhail type traps baited with the three-component lure Biolure (Unipack by Suterra®) containing ammonium acetate, trimethylamine and putrescine, were placed in the above-mentioned four locations (Table 1). Four areas located at different elevations were selected (Fig. 6): (a) Kato Lechonia (3–5 m), (b) Paleokastro (198–217 m, (c) Agios Vlasis (331–345 m), (d) Agios Georgios (612–630 m). Orchards where traps placed were characterized by mixed fruit cultivation type including several important hosts such as citrus fruits (bitter oranges, mandarins, sweet oranges), pome fruits (apples, pears, quinces) and stone fruits (apricots, peaches, plums). Traps placed at a distance further than 30 m between sampling sites, approximately 3–5 traps/ha, except of the higher elevation that the selected orchard was limited to 0.5 ha and we placed 5 traps in 0.5 ha. Detailed description of farms used is provided in Supplementary Information (Table S3).

**Table 1:** Locations of four areas in Pelion Magnesia Greece, where the McPhail type-traps for monitoring *Ceratitis capitata* population were placed.

Location	Elevation (m)	Lat. (North)	Long. (East)	Total number of traps
Kato Lechonia	5	39°19′50.21"	23° 2′19.22″	10
Paleokastro	200	39°20′25.00"	23° 3′39.75″	7
Agios Vlasis	350	39°20′29.42"	23° 4′2.19″	7
Agios Georgios	600	39°20′35.52"	23° 5′5.97″	6

#### Effect of host on adult captures (study 2)

For monitoring the seasonal and temporal population trends within the mixed fruit orchards of Kato Lechonia in the second study, we used additional 50 McPhail type traps (Tephri traps) baited with Biolure (Unipack by Suterra®) and 24 Jackson traps baited with trimedlure (Table 2)^70^. The traps established approximately at a density of ≈1–1.5 traps per hectare throughout the whole coastal area of Kato Lechonia (approximately 100 ha), without focusing on specific orchard types. Conventional, biological and abandoned farms exist in the area were included and sampled. Traps placed on citrus, pome, stone fruits and other hosts (such as figs and walnuts) located at the edges οf the orchards^44^.

**Table 2:** Hosts, number andtrap types used for monitoring *Ceratitis capitata* spatial and temporal dynamics in Kato Lechonia, Magnesia, Greece during 2008.

Trap type	Host type				Number of traps
	Citrus	Stone	Pome	Other	
Tephri	13	15	18	4	50
Jackson	7	5	8	4	24

### Data analyses

We calculated adult captures per trap per day (Flies/Trap/Day, FTD), the mean and the total number of flies captured during the season among all treatments in both studies. To depict the effect of (a) elevation and date of capture in the first study and (b) host, date of capture and temperature in the second one, on adult detection probability in both experimental approaches, we calculated, for each trap, the probability of capturing at least one adult for each observation date.

For the first study, three different Generalized Estimating Equation models (GEEs) using the negative binomial distribution were used to assess the effect of capture date, and elevation (independent variables) on the a) total number, b) male and c) female fly captures (response variables). GEE models are used for modelling longitudinal data (repeated measurements) as they take into account the within-subject dependencies (here, repeated measurements for each trap)^71^. Moreover, an event history diagram commonly used in demographic studies was created to depict the seasonal pattern of *C. capitata* captures in the four different elevations. Each row represents one trap. Squares across rows represent one week and are colored in a gradient from green (0 captures) to yellow (1–20 captures) and red (> 20 captures) according to the number captured adults. Generalized linear models (GLMs) with binomial distribution were applied to portray the effect of elevation (5, 200, 350, 600 m) on the probability of detecting an adult.

For the second study, two different GEE models using the negative binomial distribution were used to investigate the effect temperature and host (prunes, pomes, citrus and others), (independent variables) on the total number of fly captures (response variable) at different trap types, (a) Tephri and (b) Jackson. Generalized Linear Models (GLMs) with binomial distribution were applied to portray the effect of host (prunes, pomes, citrus and others) and trap type on the probability of detecting an adult, followed by post hoc test (Fisher’s Least Significance Difference) to assess the within host differences.

Parameter estimates for the GEE negative binomial models were presented as Incidence Rate Ratios (IRR) with 95% confidence intervals (CI), which is the ratio of the number of captures in a group of interest to the number of captures of the group used as reference. IRRs higher than 1 indicate more trap captures for the group of interest while IRRs lower than 1 indicate more trap captures for the reference group. Parameter estimates for the binomial GLMs were presented as Odds Ratios (OR) with 95% confidence intervals (CI).

Loess curves were used to explore capture change over time. The K-Fold cross validation was employed to identify the best fit with the lowest Root Mean Square Error (RMSE). Package ` caret ` in R was used for the K-Fold cross validation^72^.

R v4.1.2 (R Core Team 2021, R Foundation of Statistical Computing, Vienna, Austria) in RStudio v2021.09.0 (Rstudio 2021, R Foundation of Statistical Computing, Vienna, Austria) were used for the data analysis and graphical representation (ggplot2)^73^, of the results. However, in cases where models could not be estimated with R (i.e. GEEs using the negative binomial model), SPSS v29.0 (IBM SPSS Statistics for Windows, IBM Corp, Armonk, NY) was used.

### Supplementary Information

Supplementary Information.

## Data Availability

Data are available within supplementary materials file.
