# Supplementary material for: Mediterranean fruit fly population phenological patterns are strongly affected by elevation and host presence
Source: Sci Rep. 2024 Mar 12;14:6010. doi: 10.1038/s41598-024-56714-x (PMC10933347; doi:10.1038/s41598-024-56714-x)
Supplement: Supplementary file 1 — Supplementary Information. [file 41598_2024_56714_MOESM1_ESM.docx]

# **Supplementary materials**

## S1. Climatic Data

Climatic data (mean daily temperature and precipitation) for the experimental area were obtained from the Greek national database “Meteo” (<http://meteosearch.meteo.gr/>) referring to the city of Volos. The climatic profile of Volos is quite similar to Kato Lechonia, the lowest-elevation location of our study, which are both coastal and located at a short distance from each other (6.5 km north-west of Kato Lechonia). Daily maximum, minimum and average temperature and precipitation for the area of Kato Lechonia during 2008 are given in Figure S1 and Figure S2.


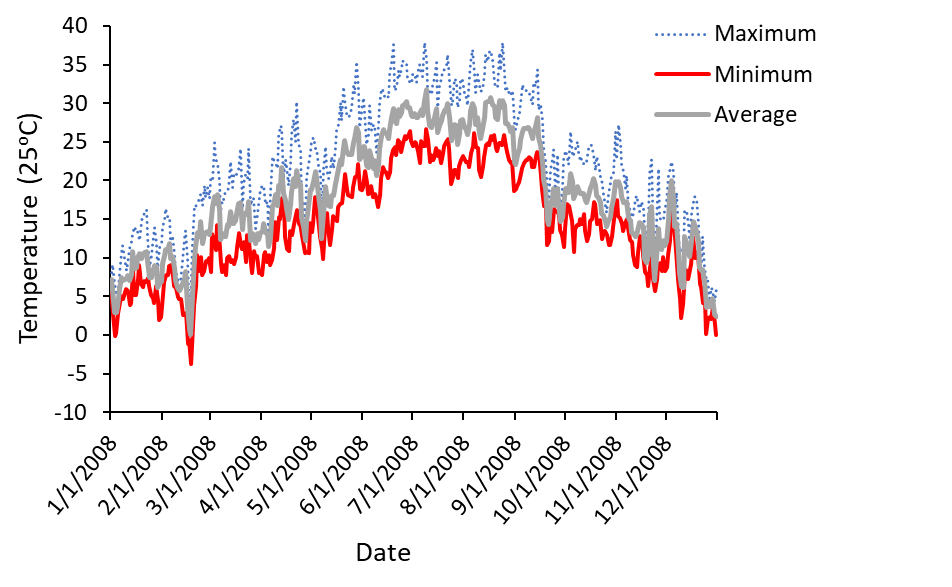


**Figure S1**: Seasonal patterns of daily maximum, minimum and average temperature in Kato Lechonia during 2008


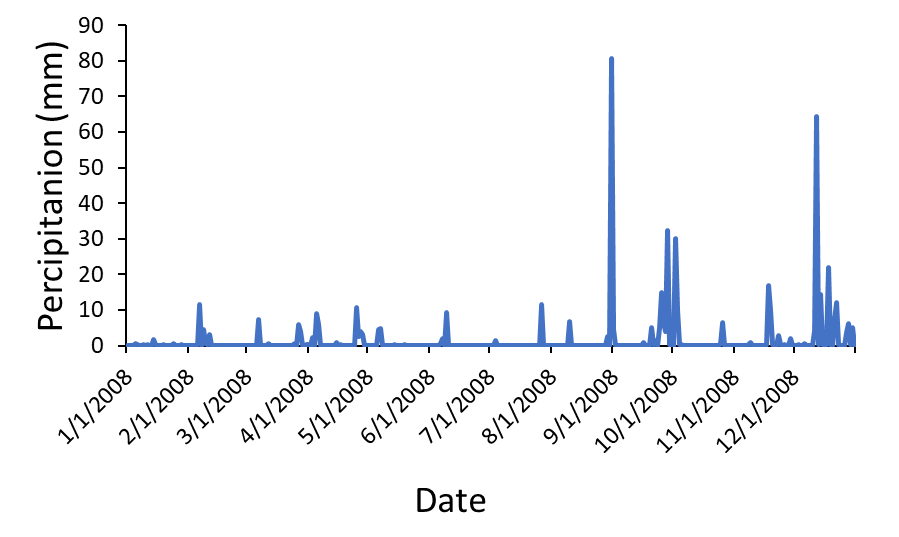


**Figure S2**: Seasonal patterns of daily precipitation levels in Kato Lechonia during 2008

## S.2. Results and Statistics- Tables

**Table S1**: Results of the generalized estimating equations (GEEs) with significant effects of elevation on the *Ceratitis capitata* adult captures

| **Dependent variable** | **Factor** | **Wald Chi-Square (df)** | **IRR (95%CI)** | ***p*** |
| --- | --- | --- | --- | --- |
| Total captures | Elevation (m) | 134.367 (1) | 0.996 (0.995, 0.997) | <0.001 |
| Males | Elevation (m) | 108.050 (1) | 0.996 (0.995, 0.997) | <0.001 |
| Females | Elevation (m) | 122.868 (1) | 0.996 (0.995, 0.996) | <0.001 |

**Table S2**: Effect of elevation on captures of *Ceratitis capitata* adults in the slopes of mount Pelion, Greece during 2008.

| **Elevation** | **Lat. (North)** | **Long. (East)** | **Traps, no** | **FTD ± SE** | | | **Mean adult captures per trap ± SE through the season** | | |
| --- | --- | --- | --- | --- | --- | --- | --- | --- | --- |
|  |  |  |  | **Males** | **Females** | **Total** | **Males** | **Females** | **Total** |
| 5 | 39 19' 12.5'' | 23 01' 38.6'' | 10 | 0.68 ± 0.12 | 2.09 ± 0.40 | 2.76 ± 0.51 | 146.50 ± 12.68 | 452.50 ± 49.53 | 599.00 ± 59.79 |
| 200 | 39 20' 15'' | 23 04' 07'' | 7 | 0.18 ± 0.06 | 0.51 ± 0.15 | 0.69 ± 0.20 | 38.57 ± 15.20 | 110.86 ± 26.99 | 149.43 ± 41.81 |
| 350 | 39 20' 37'' | 23 04' 11'' | 7 | 0.14 ± 0.03 | 0.47 ± 0.11 | 0.61 ± 0.13 | 31.00 ± 7.69 | 101.00 ± 24.18 | 132.00 ± 31.65 |
| 600 | 39 20' 39'' | 23 05' 07'' | 6 | 0.05 ± 0.02 | 0.13 ± 0.04 | 0.18 ± 0.06 | 10.67 ± 2.15 | 28.33 ± 5.56 | 39.00 ± 7.39 |
| Total |  |  | 30 | 0.26 ± 0.04 | 0.80 ± 0.13 | 1.06 ± 0.17 | 67.20 ± 11.93 | 205.93 ± 37.36 | 273.13 ± 48.85 |

**Table S3**: Detailed information describing the for farms profile used for population monitoring at different elevations

| **Elevation** | **Size (ha)** | **Hosts** | **Planting frame (m)** | **Pest Management Control** |
| --- | --- | --- | --- | --- |
|  |  |  |  |  |
| 5 | 2.0 | *Citrus* spp., *Cydonia oblonga, Ficus carica, Malus domestica, Prunus armeniaca*, *P. avium, P. domestica, P. persica, Pyrus communis* | 4x4 | Biological |
| 200 | 2.0 | *Cydonia oblonga, Prunus armeniaca*, *P. domestica, P. persica, Pyrus communis* | 4x4 | Conventional |
| 350 | 1.5 | *P. avium, P. domestica, P. persica, P. persica var. nucopersica* | 4x4 | Conventional |
| 600 | 0.5 | *Cydonia oblonga, Malus domestica, Prunus armeniaca*, *P. avium* | 4x4 | Conventional |

**Table S4**: Results of the generalized estimating equations models (GEEs) with significant effects of host and temperature on the *Ceratitis capitata* adult captures

| **Factor** | **IRR (95%CI)** | ***p*** |
| --- | --- | --- |
| **Jackson** |  |  |
| Intercept | 1.186 (0.342, 4.118) | 0.788 |
| Host (ref: prune) |  |  |
| citrus | 3.408 (0.691, 16.820) | 0.132 |
| other | 0.020 (0.004, 0.100) | <0.001 |
| pome | 1.497 (0.041, 55.214) | 0.827 |
| Temperature | 1.054 (1.016, 1.093) | 0.005 |
| Host*Temperature |  |  |
| Citrus * Temperature | 1.037 (0.986, 1.089) | 0.157 |
| Other * Temperature | 1.147 (1.094, 1.202) | <0.001 |
| Pome * Temperature | 0.973 (0.853, 1.110) | 0.680 |
| **Tephri** |  |  |
| Intercept | 2.710 (0.799, 9.187) | 0.110 |
| Host (ref: prune) |  |  |
| citrus | 2.019 (0.468, 8.721) | 0.346 |
| other | 0.052 (0.014, 0.199) | <0.001 |
| pome | 3.215 (0.704, 14.679) | 0.132 |
| Temperature | 1.077 (1.015, 1.143) | 0.014 |
| Host*Temperature |  |  |
| Citrus * Temperature | 1.001 (0.935, 1.070) | 0.986 |
| Other * Temperature | 1.120 (1.051, 1.194) | <0.001 |
| Pome * Temperature | 0.948 (0.886, 1.014) | 0.118 |

**Table S5**: Effect of different hosts and traps on mean total captures and captured flies per trap per day (FTD) of *Ceratitis capitata* adults in mount Pelion, Greece during 2008.

| **Host** | **No traps** | **FTD ± SE** | | | **Captures/trap ± SE** | | |
| --- | --- | --- | --- | --- | --- | --- | --- |
|  |  | **Males** | **Females** | **Total** | **Males** | **Females** | **Total** |
| **Tephri** | | | | | | | |
| Prune | 15 | 0.97 ± 0.23 | 1.58 ± 0.33 | 2.54 ± 0.56 | 6.79 ± 0.97 | 11.04 ± 1.32 | 17.77 ± 2.24 |
| Pome | 18 | 0.73 ± 0.15 | 1.34 ± 0.26 | 2.07 ± 0.41 | 5.14 ± 0.51 | 9.35 ± 0.83 | 14.46 ± 1.27 |
| Citrus | 13 | 2.08 ± 0.36 | 3.16 ± 0.53 | 5.23 ± 0.87 | 14.60 ± 1.64 | 22.26 ± 2.17 | 36.59 ± 3.63 |
| Other | 4 | 1.03 ± 0.28 | 1.80 ± 0.42 | 2.79 ± 0.69 | 7.20 ± 2.10 | 12.59 ± 3.02 | 19.56 ± 4.99 |
| Total | 50 | 1.20 ± 0.14 | 1.97 ± 0.21 | 3.16 ± 0.35 | 8.26 ± 0.58 | 13.46 ± 0.80 | 21.61 ± 1.34 |
| **Jackson** | | | | | | | |
| Prune | 5 | 0.63 ± 0.16 | 0.0 | 0.63 ± 0.16 | 4.43 ± 0.97 | 0.0 | 4.43 ± 0.97 |
| Pome | 8 | 0.47 ± 0.12 | 0.0 | 0.47 ± 0.12 | 3.30 ± 0.73 | 0.0 | 3.30 ± 0.73 |
| Citrus | 7 | 5.41 ± 1.56 | 0.0 | 5.41 ± 1.56 | 37.89 ± 0.72 | 0.0 | 37.89 ± 0.72 |
| Other | 4 | 0.48 ± 0.14 | 0.0 | 0.48 ± 0.14 | 3.38 ± 0.73 | 0.0 | 3.38 ± 0.73 |
| Total | 24 | 1.75 ± 0.45 | 0.0 | 1.75 ± 0.45 | 13.63 ± 2.28 | 0.0 | 13.63 ± 2.28 |

**Table S6:** Pairwise comparisons of total fly captures between the different hosts adjusted for temperature per trap type.

| **Jackson** |  |  |  |  |
| --- | --- | --- | --- | --- |
|  | Citrus | Pome | Prune | Other |
| Citrus | - | 0.007 | 0.010 | 0.006 |
| Pome |  | - | 0.568 | 0.599 |
| Prune |  |  | - | 0.262 |
| Other |  |  |  | - |
| **Tephri** |  |  |  |  |
|  | Citrus | Pome | Prune | Other |
| Citrus | - | 0.011 | 0.044 | 0.033 |
| Pome |  | - | 0.654 | 0.989 |
| Prune |  |  | - | 0.740 |
| Other |  |  |  | - |


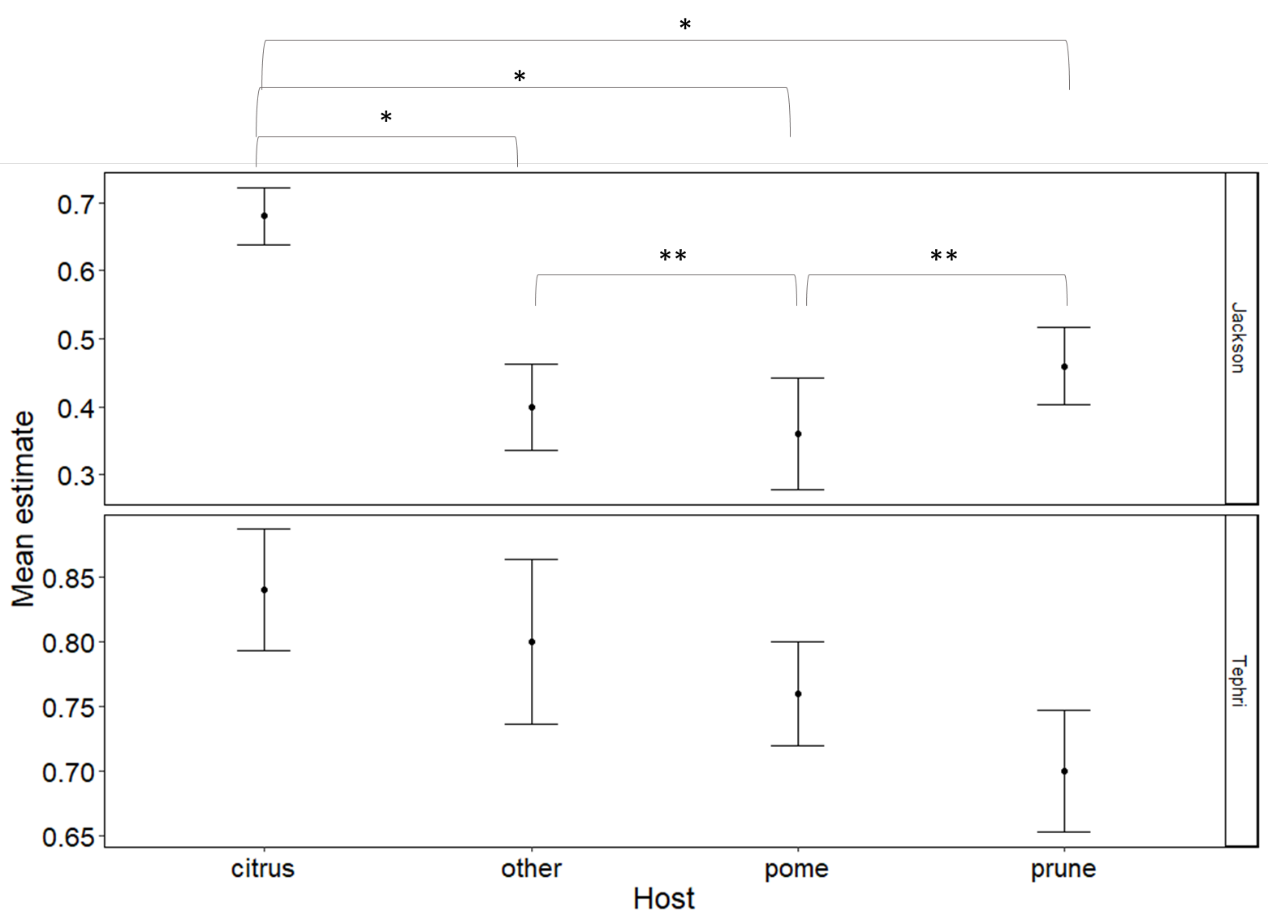
Figure S3: Mean estimates (SE) from the Negative binomial GEE model for the different trap types
